# Effect of Therapy Ball Seating on Learning and Sitting Discomforts among Saudi Female Students

**DOI:** 10.1155/2013/153165

**Published:** 2013-06-10

**Authors:** Einas Al-Eisa, Syamala Buragadda, Ganeswara Rao Melam

**Affiliations:** ^1^Female Centre for Science and Medical Studies, King Saud University, Riyadh, Saudi Arabia; ^2^Department of Rehabilitation Sciences, College of Applied Medical Sciences, King Saud University, P.O. Box 10219, Riyadh 11433, Saudi Arabia

## Abstract

The aim of the study was to evaluate the effect of therapy ball seating as an alternative for typical chair seating in a classroom. We evaluated the effect of ball seating on the student's sitting discomfort and academic performance using Cornell Musculoskeletal Discomfort Questionnaire and problem-based learning scales, respectively. A sample of convenience was taken. Data was collected and analyzed using *t*-test. Subjects experienced a major discomfort at neck and a minor discomfort at knee joint. Results showed that there was a significant improvement (*P* ≤ 0.05) in sitting discomfort and student's performance when seated on therapy balls compared to typical classroom chairs. This study provides evidence for the effectiveness of therapy balls as a classroom seating for students who exhibit sitting discomfort and problem-based learning.

## 1. Introduction 

Factors like student's gender, personality differences, and class room environment affects the student's learning and participation in the classroom. Most of the college and university professors follow a lecture method of teaching which involves delivering a lecture where the instructor speaks and the student sits for a prolonged period of time in the classroom. Human brain maintains an optimal state of arousal and attention only with the help of sensory stimuli, and arousal can be either low associated with lethargy and drowsiness or high leading to hyperactivity and distractibility. Sensory modulation is required for optimal attention and learning [1, 2]. 

It was assumed that the student's capacity to pay attention depends on the ability to access learning opportunities at school/university [3]. Instructors/teachers usually adopt various behavioral programs to improve the student's in-seat behavior [4] Behavioral differences were observed when students were seated in front row compared to middle- and back-row seating. They found that students were more attentive when seated in the front rows [5, 6]. A study conducted on autistic children of age three to four, resulted in the improvement of classroom behavior when they were asked to sit on therapy balls instead of a typical classroom chair [7]. Another study on fourth grade classroom seating showed that the students had better in-seat behavior and legible word productivity when they sat on therapy balls. Survey completed by the teacher and the students indicated the preference for therapy ball seating [8]. 

Researchers found that the design of the class should be flexible, creative, and problem solving. Cornell defined furniture as both a tool and an environment. To create a suitable learning environment for students it is mandatory to think of the furniture and seating arrangements of a classroom [8]. Some researchers also reviewed different seating arrangements in terms of rows and columns in relation to student's interaction and specific interaction patterns [9–12]. Experimentally controlled research on the use of therapy balls as an alternative seating arrangement for children with attention and hyperactivity concerns was done. However, limited literature is available on the use of therapy balls as alternative class room seating in normal students. 

Since 1991, the ball had replaced chairs in schools for thousands of children of Europe due to increased information on the postural benefits of “active sitting” and back injury prevention. The classroom teachers found that (a) hyperactive children became calmer and could focus for longer periods (b) other children could generally concentrate better, (c) handwriting skills improved for children with poor penmanship, (d) children often showed a better understanding of subject material, and (e) disorganized children developed a better sense of organization [13]. Swiss balls have become increasingly popular as an alternative to office chairs as they help to reduce the prevalence of low-back pain, engage the abdominal and back muscles, and maintain proper posture to remain balanced on the ball. This is sometimes prescribed by physical therapists for back-pain patients [14]. 

Some authors concluded that discomfort is highly related to sitting on chairs [15]. Kee and Karwowski suggested that static postures maintained for 60 seconds cause greater discomfort for the hip joint and less discomfort for the elbow joint [16]. However, any static posture for a prolonged time can cause discomfort, pain, injury, and reduction of efficiency. Sitting discomfort is traditionally evaluated with subjective rating scale such as general comfort rating, body area comfort rating, chair feature checklist, and direct ranking of chairs [17]. On the other hand, Fenety et al. adopted an interface pressure mat to continuously record in-chair movement as an indirect measurement of sitting discomfort [18]. 

Problem-based learning scale is an assessment tool to measure 4 areas of competency. An instructor rates each student's performance on the domains like (1) participation and communication, (2) cooperation/team building skills, (3) comprehension/reasoning skills, and (4) knowledge/information gathering skills [19].

The purpose of our study was to evaluate the effect of therapy ball seating as an alternative for typical chair seating on sitting discomfort and learning in a classroom. 

## 2. Materials and Methods 

A sample of convenience was used which included 40 healthy female physical therapy students from college of applied medical sciences, King Saud university, Saudi Arabia. Study procedures were approved by the ethical committee of the university, and an informed consent was obtained from all the volunteers. Subjects with a history of recent injury, any medical problem of low-back pain, pregnant students, and students wearing high heels were excluded from the study. Subject's weight and height were recorded, and the mean age was 20 ± 2 years with a BMI 24 ± 3 kg/m^2^. Student's discomfort was self-rated using Cornell Musculoskeletal Discomfort Questionnaire which determines the site and severity of pain. The scores were measured as follows.

 Frequency score: never = 0; 1-2 times/week = 1.5; 3-4 times/week = 3.5; once/day = 5; several times/day = 10.
 Discomfort score = 1—mild; 2—moderate; 3—severe.
 Interference score = 1—mild; 2—moderate; 3—severe.
 Multiplying the above frequency score (0, 1.5, 3.5, 5, 10) by the discomfort score (1, 2, 3) and the interference score (1, 2, 3) gives the discomfort score.

The instructor evaluated student's performance with problem based-learning (PBL) scale (Table 1) [19]. 

*Procedure*.  A classic firm therapy ball made of PVC base with burst and loading rates of 285 and 1000 lbs, respectively, was selected. The size of the ball ranged from 45 to 75 centimeters.

Swiss balls were selected based on their height and weight, and each ball was labeled with the participant's name. They were instructed to sit with trunk extended, hip and knee flexed to 90°, and feet resting on the floor. A table was placed in front of each student to support their arms while writing. For the first 4 weeks participants attended 3 lectures per week while seated in their regular class room chairs and were asked to evaluate their sitting discomfort using Cornell Musculoskeletal Discomfort Questionnaire at the end of the 4th week. The instructor also evaluated each student's performance using problem-based learning (PBL) scale. 

For the next 4 weeks the students were instructed to sit on their Swiss balls (3 lectures per week). The lecture was divided into 2 sessions, each with a duration of 50 minutes with 10 minutes break in between the sessions. At the end of the 4th week students were asked to rate their discomfort using Cornell Musculoskeletal Discomfort Questionnaire, and the performance was evaluated by the instructor using problem-based learning (PBL) scale. 

## 3. Results 

Data was analyzed using SPSS software. Means and standard deviations were calculated. Paired *t*-test was used to analyze the discomfort and PBL scores of chair and ball seating. The level of significance was set at *P* ≤ 0.05. 

The PBL score was high in all four domains (participation, comprehension, cooperation, and knowledge) when students sat on the Swiss ball compared to typical chair seating. On the other hand, sitting discomfort scores dropped significantly (*P* < 0.05) when the subjects were seated on Swiss balls (Tables 2 and 3). After using therapy balls neck discomfort was reduced significantly when compared to other regions of the body, whereas subjects experienced knee discomfort.

## 4. Discussion 

Students experienced greater discomfort at neck, followed by shoulder, low back, hip, and knee regions. The results of the study showed that there was a significant improvement in sitting discomfort and attention in students with therapy ball seating. This trend of increased in-seat behavior correlates with the findings of the study conducted by Schilling and Schwartz [7]. They concluded that children with autism showed a better in- and out-seat behavior when placed on therapy balls. 

The results of our study correlates with the study done by Schilling et al. where children with attention deficit hyperactivity concerns improved their inclusive educational practice and interdisciplinary learning when therapy balls were used as an alternative seating [20]. Both physical therapy and occupational therapy disciplines primarily explain sensory processing theory which explains that “brain handles sensory information to enable person's attention in occupation” [21]. It was concluded that Swiss ball is an excellent alternative to chair in improving sitting discomfort especially on the neck, shoulder, and lower-back regions. This supports the findings of Gregory, Dunk and Callaghan who stated that sitting on the ball help in reducing the prevalence of low-back pain by engaging the abdominal and back muscles in maintaining proper posture to remain balanced on the ball [14]. In addition, it was documented in the literature that sitting discomfort is highly related to chair seating [15, 16]. McGill et al. assessed torso muscle activation using EMG in subjects sitting for 30 min on an exercise ball and 30 min in an office chair and a 3-dimensional lumbar position was recorded for every 5 minutes. This data was inserted to a series of biomechanical models to calculate a measure of L4-L5 compression and spine stability. They found no differences between a chair and a Swiss-ball seating in muscle activation, spine posture, spine loads, or overall spine stability; instead, sitting on a ball appears to spread out the contact area resulting in an uncomfortable soft tissue compression causing discomfort [22]. A study was done on 20 healthy male subjects to measure perceived discomforts at varying joint motions in sitting and standing postures. Three ranking systems were developed based on perceived discomfort, and they concluded that hip and back motions have higher discomfort ratings than any other joint motions [23]. In our study we found that the neck exhibited higher discomfort than any other body area.

Another study investigated the joint angles of isocomfort (JAI) for female subjects in a 60-second static standing posture and found that hip postures are more stressful and JAI for females were significantly different than those for males [24].

## 5. Conclusion 

Study results concluded that sitting discomfort and performance of students improved while seating on therapy balls when compared to typical classroom chair seating. Although discomfort was self-rated, our results granted the opportunity to replace typical classroom chairs with therapy balls, and future research should be performed to determine similar or even stronger evidence in socioeconomic, ethnic and gender differences in a larger group of randomized population.

## Figures and Tables

**Table 1:** The criteria guidelines for evaluation using problem-based learning (PBL) scale.

Scores	Participation and communication	Cooperation/team building skills	Comprehension/reasoning skills	Knowledge/information gathering skills
1	Does not speak to others Does not respond to verbal/nonverbal cues.	Unwilling to take up any task Does not contribute to identify learning issues.	Does not demonstrate understanding/clarification of learning concepts.	Dose not prepare for the session.

2	Responds to verbal cues and rarely asks questions.	Rarely participates and takes up the task only when asked.	Understands of tasks only under considerable guidance.	Prepares only for certain issues.

3	Responds to both verbal/nonverbal cues Occasionally asks questions.	Participation in most learning cases.	Understanding of tasks under little guidance.	Preparation for most learning issues.

4	Regularly asks questions and present the ideas clearly.	Participates regularly and encourages others to participate.	Clearly understands of the concepts and draws valid conclusions.	Prepares well and recognizes integration of knowledge when explained by others.

5	Demonstres of listening and summarization skills and is able to lead the discussion of a group	Organizes a group and can make others to actively participate in group activities	Explains concepts clearly and can find flaws in the data with good reasoning	Prepares well for the sessions by identifying key references.

**Table 2:** Cornell Musculoskeletal Discomfort Questionnaire scores of subjects seated on chair and ball.

	Neck		Shoulder		Low back		Hip		Knee	
	Chair	Ball	Chair	Ball	Chair	Ball	Chair	Ball	Chair	Ball
Mean	28.3	2.4	22.6	0.6	10.3	1.40	1.2	0.2	0	0.3
SD	4.98	1.48	5.77	0.5	8.2	0.85	0.6	0.6	0	0.9
SE	0.78	0.23	0.91	0.11	1.29	0.13	0.09	0.1	0	0.14
*t*-value	2.1		1.25		1.30		1.62		0.02	

*P*< 0.05 level of significance										

SD: standard deviation; SE: standard error; *t*-value: *t*-test scores.

**Table 3:** Problem-based learning scale (PBL) scores of subjects seated on chair and ball.

	Participation		Comprehension		Cooperation		Knowledge	
	Chair	Ball	Chair	Ball	Chair	Ball	Chair	Ball
Mean	3.2	3.8	2.9	3.6	3	3.7	2.9	3.6
SD	0.4	0.9	0.37	0.54	0	0.8	0.37	0.54
SE	0.06	0.15	0.05	0.08	0	0.12	0.05	0.08
*t*-value	0.00		1.6		1.85		1.6	

*P*< 0.05 level of significance								

SD: standard deviation; SE: standard error; *t*-value: *t*-test scores.
